# Segment-Resolved Gas Concentration Measurements by a Time Domain Multiplexed Dual Comb Method

**DOI:** 10.3390/s20061566

**Published:** 2020-03-11

**Authors:** Xinyi Chen, Weipeng Zhang, Yujia Zhang, Minjian Lu, Yan Li, Haoyun Wei

**Affiliations:** 1 State Key Laboratory of Precision Measurement Technology & Instruments, Department of Precision Instrument, Tsinghua University, Beijing 100084, China; chen-xy17@mails.tsinghua.edu.cn (X.C.); weipengz@princeton.edu (W.Z.); zhang-yj18@mails.tsinghua.edu.cn (Y.Z.); lumj19@mails.tsinghua.edu.cn (M.L.); liyan@mail.tsinghua.edu.cn (Y.L.); 2 Department of Electrical Engineering, Princeton University, Princeton, NJ 08544, USA

**Keywords:** absorption spectrum, dual-comb spectroscopy, gas concentration measurement

## Abstract

Locating gas concentration changes in widespread locations can be conducive to environmental atmospheric detection, gas emissions monitoring, production process control, etc. A time domain multiplexed dual-comb system for segment-resolved gas concentration measurement is reported in this work. Both absorption spectra and path lengths for multiple path-segments in a target path can be derived from the time domain separated interferograms and then the equivalent gas concentrations in each segment can be retrieved separately. A benchtop experiment aiming at a target path with three path-segments of different gases has been demonstrated. The relative deviation of gas concentration retrieval is 1.08% in 1 s. Besides, additional numerical simulations prove that the crosstalk between the interference signals affects the spectrum analysis by no more than 0.1% for a kilometer-long atmospheric absorption detection. Therefore, achieving a gridded measurement of regional gas concentration in the open air can be foreseen using this method.

## 1. Introduction

Gas remote sensing, as a vital tool, plays an irreplaceable role in many fields, such as environmental monitoring, industrial process control, public safety, etc. A variety of optical spectroscopic methods have been demonstrated to enable gas remote sensing, such as tunable diode laser absorption spectroscopy (TDLAS) [1,2], differential optical absorption spectroscopy (DOAS) [3,4], Fourier transform spectroscopy (FTS) [5,6], and dual-comb spectroscopy (DCS) [7,8,9,10,11]. Among them, the DCS method has triggered quantum leap advancements [12,13,14,15,16,17,18] due to its fantastic potential of broad spectral coverage [19,20], high resolution [21,22] and high detection speed without any moving elements. The ms-level refreshment rate of dual-comb method makes the influence of turbulence frozen in a single measurement, supporting the use of DCS for long open-path applications such as pollutant monitoring [23] and urban CO_2_ emission estimation [24]. 

Although the feasibility of the dual-comb remote sensing method in various situations has been verified, current research is still limited to the gas concentration detection of a single long path. In actual application, it is also important to locate the concentration changes over a target path by segmental measurements, which can provide more effective information for gridding analysis and dynamic control. This requires simultaneous determination of the absorption spectra and segment lengths according to the Lambert-Beer law. The dual-comb method coincidently has been demonstrated in spectral measurement or ranging [25,26,27]. By combining these two capacities of the dual-comb method, it could make gridded quantitative spectroscopic analyses possible in open-path remote sensing.

In this paper, we demonstrate a gas distribution measurement scheme using one detector based on the dual-comb method. Multiple interferograms (IGMs) generated by corresponding segments in the target path were interleaved in a single dual-comb sampling period. Both absorption spectra and segment lengths can be derived from these time domain separated IGMs and then the concentrations of gases in each segment deduced. The effects on spectrum analysis introduced by signal crosstalk have been discussed, which proves that the absorption information of different segments can be well distinguished. This segment-resolved gas sensing method can provide effective criteria for the locating of gas distribution especially for regional open-path measurements.

## 2. Method

**Figure 1:**
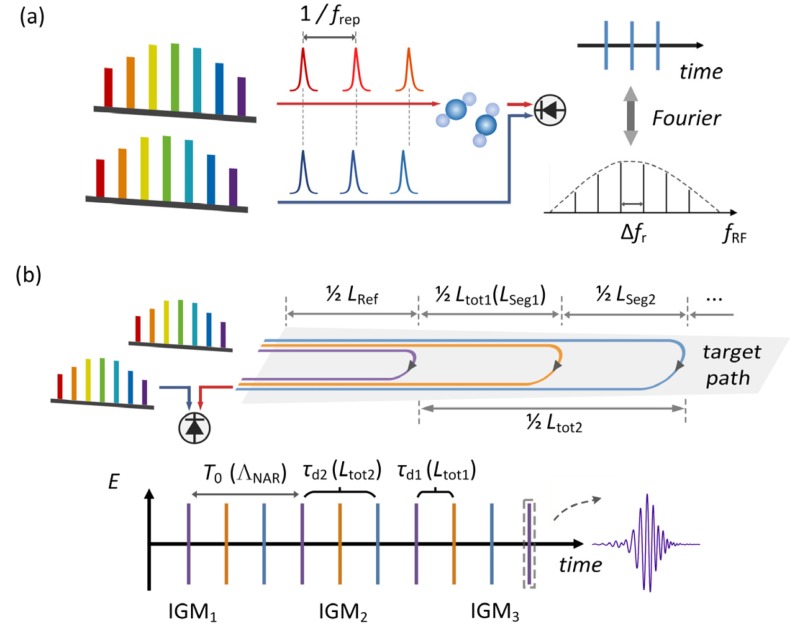
Schematic diagram of dual-comb method and time domain multiplexed dual-comb method. (**a**) Dual-comb asynchronous sampling process and the interference signal in the time domain and the RF domain. *f*_rep,_ the repetition rate of the comb. Δ*f*_r_ is the difference of *f*_rep_ between the two combs. (**b**) Scenario diagram of the time domain multiplexed dual-comb gas distribution measurement scheme and the interleaved IGMs marked with the corresponding spectral and length information.

The principle of dual-comb method is well known as it utilizes asynchronous optical sampling to obtain multi-heterodyne interference signal and converts the optical spectrum into the detectable radio frequency (RF) spectrum with no need for dispersion or interference optical elements, as shown in Figure 1a. The absorption spectrum of the samples in the beam path can be derived from the interference signal by applying a Fourier transform and frequency remapping. Thus, one can obtain millions of spectral elements with effective optical resolution of up to a few thousandths of a wavenumber (about hundred MHz), which is much higher than the requirement of the several-GHz-linewidth trace gas absorption detection and supports further sampling period exploitation. Here, we interleaved multiple IGMs in a single sampling period *T*_0_, as shown in Figure 1b, to determine the gas distribution along the target path. IGMs (IGM_1_, IGM_2_, IGM_3_…) of different colors are generated by the light signals passing through different paths. Each path consists of a different number of path-segments (Ref., Seg.1, Seg.2…) and contains different gas absorption information that can be used for segment-resolved gas concentration retrieval. In order to separate the IGMs on the time axis, beam splitters were inserted to the proper locations in the target path to introduce different optical delays relative to *T*_0_, shown as *τ*_d1_, *τ*_d2_. Optimal utilization of the sampling period can be achieved by adjusting the relative positions of these splitters.

**Figure 2:**
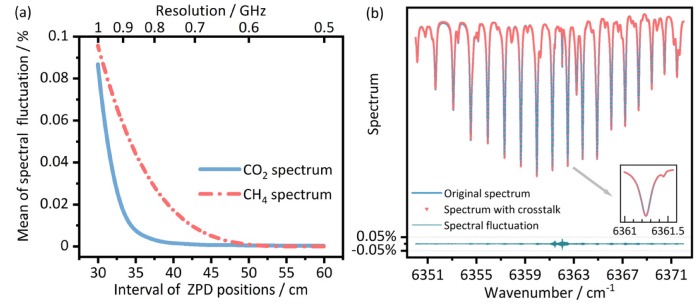
Results of the simulation for the crosstalk introduced by the superposition of IGMs. (**a**) The mean of spectral fluctuations after superposition at different ZPD position intervals. The upper horizontal axis represents the maximum resolution that can be achieved at different intervals. (**b**) A part of the CO_2_ spectrum at a ZPD position interval corresponding to 1 GHz (0.033 cm^−1^) resolution. The inset is a magnified view of the area where the spectrum has relatively large fluctuations.

To retrieve the gas concentration in each segment, both the absorption spectrum and path length for each path are required according to the Lambert-Beer law. On the one hand, for absorption spectra measurements, though these can still be derived from the separated IGMs, the thing that should be carefully inspected in the proposed scheme is the possible crosstalk in the spectra caused by the superposition of the time domain multiplexed IGMs. To evaluate the magnitude of this effect, we simulated a series of intervals between the zero path difference (ZPD) positions of the adjacent IGMs and obtained the spectral fluctuations by taking the difference between the spectrum obtained from the superposed IGM and the original one as an indicator. A typical result is shown in Figure 2a. Here, a summer mean-latitude atmospheric absorption model was adopted, in which the mixing ratio of CO_2_ and CH_4_ is 0.033% and 0.00017%, respectively, and the path length was set to be 1000 m. The ZPD interval of the adjacent IGMs was selected to be 30–60 cm, which corresponded to four to two IGMs interleaved in a single sampling period when *f*_rep_ was 250 MHz. At each interval, we took out ZPD centered data with a full length of corresponding interval for analysis. The achievable maximum resolution is shown in the upper horizontal axis. It can be seen that the introduced crosstalk in the spectrum by this method is no more than 0.1% for both CO_2_ (blue) and CH_4_ (red). The different changing trends of spectral fluctuations are essential, due to the differences of their absorption lines parameters in this spectral region and the difference of their concentrations. A more visual comparison can be found in Figure 2b. Here, The ZPD interval is equal to 30 cm corresponding to 1 GHz (0.033 cm^−1^) resolution. The spectrum in the proposed time domain multiplexed scheme has a slight difference (less than 0.1%) than that in the normal scheme. This crosstalk is typically smaller than the measurement signal-to-noise ratio (SNR) and can thus be ignored. Essentially, this crosstalk is determined by the ratio of the linewidth of the gas absorption to the measurement resolution of the multiplexed scheme. The linewidth is limited by the free induction decay and broadened by self- and environmental effects. The resolution that can be achieved is determined by the ZPD interval. Careful trade-off is needed before applying the time domain multiplexed scheme. For gas sensing in normal temperature and pressure conditions, 1 GHz resolution can well resolve most of the environment-broadened absorption lines of trace gases. So at least 4 IGMs can be multiplexed for the case where *f*_rep_ equals 250 MHz, or 10 IGMs for where *f*_rep_ equals 100 MHz with the same ZPD interval. Larger multiplexing number for finer segmented measurement can be further achieved with compromised resolution or lower *f*_rep_. 

On the other hand, the path length for each segment should be measured in the proposed scheme. The optical path difference between the reference and measurement path can be calculated based on the time of flight (TOF) method for dual-comb ranging by using the temporal interval between the envelope centers of the IGMs. No further phase retrieval is required as the measurement uncertainty of path length by TOF method is typically well enough for the proposed scheme. The path length (*L*_tot_) can be derived from the measured time delay (*τ*_d_) as shown in Figure 1b, following the relationship *L*_tot_ *= v*_g_ × *τ*_d_ × Δ*f*_r_/*f*_rep_. Here, *τ*_d_ = Δ*D/f*_samp_. Δ*D* is the data interval between the peaks of the IGMs. *f*_samp_ is the sampling frequency. *v*_g_ is the speed of light in air. The retrieved length *L*_tot_ is limited in a non-ambiguity range (NAR, *Λ*_NAR_ = *v*_g_/*f*_rep_), which is from sub-meter to several meters for dual-comb method. For an open-path application, it is not difficult to achieve reliable NAR extension by using metadata from Global Position System (GPS) [8] or by applying synthetic wavelength method [28,29]. Finally, the path length of each segment (*L*_seg*n*_) is calculated as the difference between the adjacent paths.

The concentration of target gas in each segment can be retrieved with the knowledge of these absorption spectra and path lengths for each path. Normally, only the product of the path length and the equivalent gas concentration can be derived from one measured spectrum according to the Lambert-Beer law. Here the equivalent concentration is the retrieved concentration assuming the target gas is evenly distributed in the corresponding measurement path. Equation (1), derived from this law, reveals the relationship between the equivalent concentration *α*_tot_ in the whole path length *L*_tot_ and that in each path-segment (*α*_seg*n*_, *L*_seg*n*_, n = 1, 2, 3…) for a specific target gas. The equivalent concentrations (*α*_tot,_ *α*_seg*n*_) can be retrieved from the spectra that contain different absorption information and the corresponding path lengths using the least squares method. The segment-resolved gas concentrations *α*_seg*n*_ can be used for gas distribution analysis along the target path.

(1) $$\alpha_{\mathrm{tot}}\times L_{\mathrm{tot}}=\sum \alpha_{\mathrm{seg}\;n}\times L_{\mathrm{seg}\;n}\;(n=1,2,3\ldots )$$

## 3. Experimental Setup

**Figure 3:**
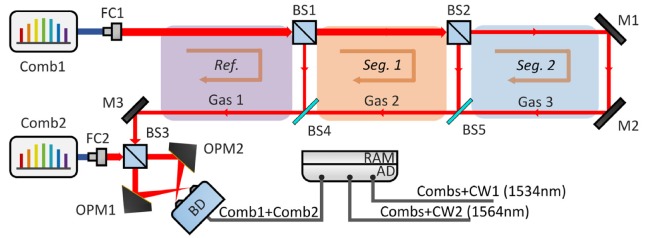
Experimental setup of the gas distribution measurement scheme. AD, analog to digital converter. BD, balance detector. BS1, BS2, BS3, cube beam splitter. BS4, BS5, pellicle beam splitter. Comb1, Comb2, optical frequency comb. CW1, CW2, continuous wave laser. FC1, FC2, fiber collimator. M1, M2, M3, mirror. OPM1, OPM2, off-axis parabolic mirror. RAM, random-access memory. Red line, space light. Blue line, fiber. Grey line, cable. Ref., reference path. Seg.1, Seg.2, different segments of the target path.

In order to verify the feasibility of the proposed scheme, we set up a conceptual distribution scenario under laboratory conditions. Three cells were used to introduce gas absorption. Figure 3 illustrates the schematic diagram of our time domain multiplexed dual-comb setup. In fact, it does not distinguish the absorptive or non-absorptive parts of the gaseous path. What can be measured in this miniature scene is the equivalent absorption path length (similar to remote sensing with inhomogeneous gas distribution) and we used it to calculate the equivalent gas concentration in each path-segment where each gas cell was located. Cells at different locations had the same nominal partial pressure (100 ± 0.2 torr) but different gas types (hydrogen cyanide, H^13^CN; acetylene, ^13^C_2_H_2_; and acetylene, ^12^C_2_H_2_). These three samples were carefully selected with significant absorptions in the detection band.

Two optical frequency combs were used as the light sources (FC-1500, Menlo Systems, Munich, Germany), which had almost the same *f*_rep_ of about 250 MHz. ∆*f*_r_ was chosen to be 290 Hz so that the interference signal generated by the entire output of the combs was within the frequency range (<*f*_rep_/2) and no aliasing occurred. Cube beam splitters and pellicle beam splitters were used to split the light. We controlled the splitting ratio (R:T, 20:80, 50:50) of the two cubes (BS1, BS2) in order that the optical signal intensity returned from each position was as close as possible. The splitting ratio of the pellicle splitter used in this band is generally close to 50:50, and it was used here to minimize the multiple reflection effects. Two off-axis parabolic mirrors were used to focus the beams onto the receiving surface of the detector and are immune to dispersion. A balance detector (1837, 300 MHz, New Focus, Irvine, CA, USA) was used to maximize the available dynamic range for the heterodyne signal by suppressing the homodyne signal from the individual channels. Two slow-loop controlled diode lasers (1534.223 nm, 1564.701 nm, Redfern Integrated Optics, Santa Clara, CA, USA) were used as the reference lasers for digital calibration [16,30,31,32]. All signals were recorded by a Field Programmable Gate Array (FPGA) (250 MS/s, 14 bits, 4 CHs). The acquisition time was greater than 1 s, which benefited from an on-board random-access memory (RAM). This large storage buffer allowed high throughput acquisition and processing. Since recording one set of IGMs required only about 3.4 ms (1/∆*f*_r_), more than 400 sets of consecutive IGMs could be obtained within a single acquisition.

## 4. Results

### 4.1. Measurement of Absorption Spectrum and Path Length

**Figure 4:**
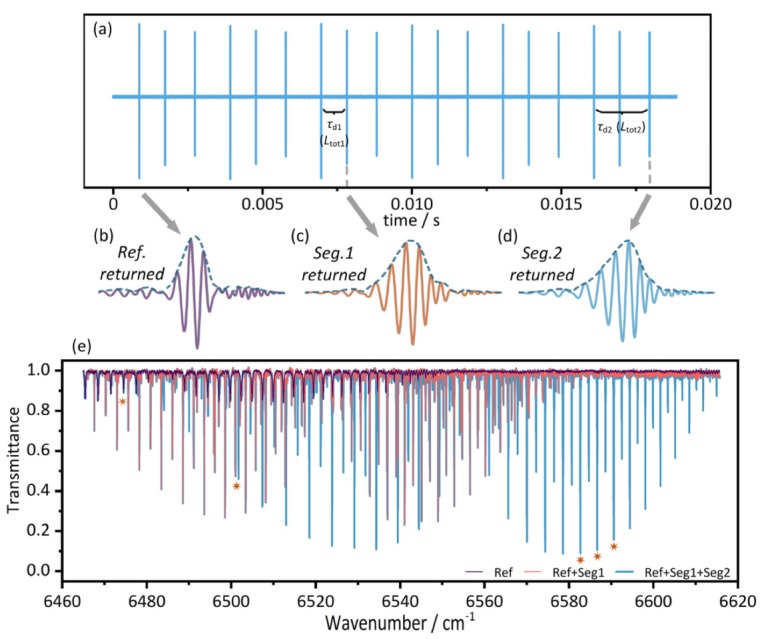
IGMs at different scales and the corresponding spectra. (**a**) Part of the measured continuous IGMs. (**b**), (**c**) and (**d**) Expanded view of the IGMs that returned from different segments (lasting 1.6 μs) and their envelopes extracted by Hilbert transform and interpolate. (**e**) Part of the transmission curves derived from corresponding IGMs.

Both absorption spectra and path lengths can be derived from the time domain separated IGMs. Figure 4 shows the IGMs at different scales. Figure 4a is a part of the continuous IGMs. Figure 4b–d is expanded views of the IGMs that returned from different segments. Absorption spectrum for each path can be obtained by performing Fourier transform on each co-added IGM. The transmittance was obtained by segmentally baseline fitting, for overcoming the sub-peaks and bumps in the non-smooth spectral baseline. No reference spectrum was used here, in order to avoid the additional baseline etalons [7]. The entire spectrum of the comb could cover numerous absorption bands of the three gases (H^13^CN, ^13^C_2_H_2_, ^12^C_2_H_2_), and the range selected to be shown in Figure 4e covers the full branch of the *v*_6_ + *v*_4_ ro-vibration of ^12^C_2_H_2_ molecule. The curves of different colors show the transmittance derived from the corresponding IGMs in Figure 4b–d.

The resolution in this measurement is 750 MHz (~0.025 cm^−1^) in the optical frequency domain. The overlap portion of the curves represents the absorption of H^13^CN and ^13^C_2_H_2_ in the reference path and Seg.1, respectively, and the non-overlapping portion of the blue curve is introduced by the absorption of ^12^C_2_H_2_ in Seg.2. There is no obvious crosstalk between the above-mentioned absorption information. The details of the areas marked with the asterisks can be found in Figure 6. Gas type and absorption intensity in each path can be distinguished from these spectral results, providing support for subsequent distribution measurement. In the case where the types of gases contained in each segment are the same, the difference in spectrum will be reflected in increasing absorption intensity rather than the different frequency components.

**Figure 5:**
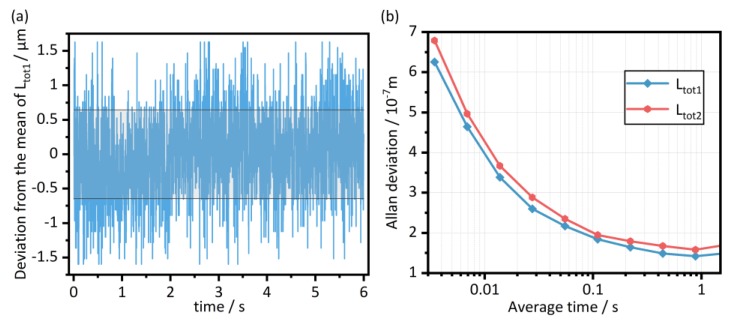
Results of the equivalent absorption path length measurement. (**a**) Measurement data of *L*_tot1_ separated at an interval of 5 min. Each set of data had a length of 1 s. The gray area represents the ± σ interval (calculated from 6 s data, σ = 0.6 μm). (**b**) Allan deviation of the path lengths measurement.

The equivalent absorption path length of each path-segment was derived from the separated IGMs by the TOF method. Figure 4b–d shows the effect of extracting the envelopes by the Hilbert transform. The envelopes then were interpolated for more accurate result of the interval Δ*D* between the adjacent peaks. Then, the optical path length of each path-segment was calculated. After that, we subtracted the optical path length of the glass portion of each corresponding path, which was calculated using the nominal component length and the nominal glass refractive index. For the gas part, it is a paradox to estimate the refractive index of the sample gas before knowing its concentration, so we ignored the slight refractive index difference between sample gases and air, and uniformly used the refractive index of air to calculate the equivalent path length. Specific path has been shown in Figure 1 and Figure 3. The measurement results of *L*_tot1_ are shown in Figure 5a without averaging. The results consist of six 1-second samples with 5-min intervals. The mean value and the standard deviation σ of *L*_tot1_ are 0.466 776 5 m and 0.6 μm, respectively. It can be seen that the results are consistently stable throughout the measurement, even if the measurement was intermittent. The Allan deviation of the measurement versus averaging time is shown in Figure 5b. Take the results of *L*_tot1_ as an example, about 0.6 μm Allan deviation was obtained without averaging. And it was improved to 0.14 μm through less than 1 s averaging. It can be seen that the magnitude of Allan deviation was improved by about five times within 1 s. This enhancement provides strong evidence that the system can maintain a stable level and the measured length can reach a relative Allan deviation less than 1 × 10^−6^ just by the TOF method within the NAR. This performance of path length measurement by dual-comb TOF method is well qualified for gas sensing and no interferometric phase retrieval is needed.

### 4.2. Gas Concentration Retrieval

**Figure 6:**
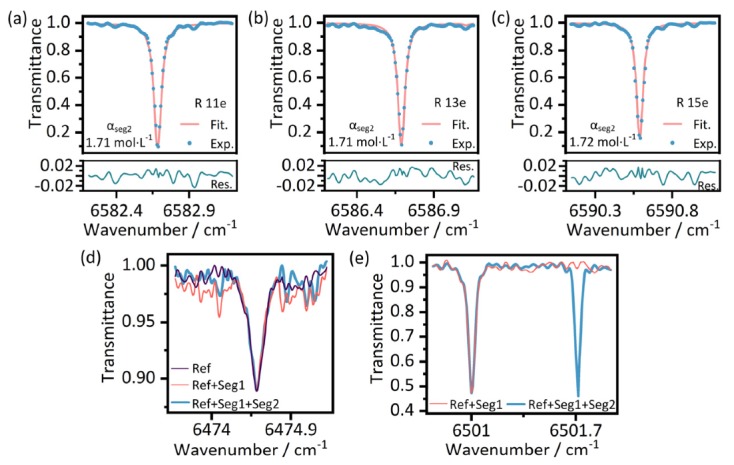
The magnified view of the absorption lines of the three gases. (**a**) (**b**) and (**c**) Three different absorption lines of ^12^C_2_H_2_ and the results of the corresponding fitting for the concentration retrieval using the least squares method. (**d**) Absorption line of H^13^CN from the three different spectra. (**e**) Absorption lines of ^13^C_2_H_2_ (left) and ^12^C_2_H_2_ (right).

The segment-resolved equivalent gas concentration of each path-segment can be retrieved separately. We take the retrieval of ^12^C_2_H_2_ concentration as an example. To do this, a transmission model with Voigt line-shape parameters was fitted to three absorption lines in *v*_6_ + *v*_4_ ro-vibration of ^12^C_2_H_2_ using the least squares method with the obtained equivalent path length. We can obtain the concentration *α*_ref*,*_ *α*_tot1*,*_ *α*_tot2_ from all sets of IGMs returned from different segments by the same fitting method. Then we can retrieve the concentration in Seg.1 *α*_seg1_ and Seg.2 *α*_seg2_ with the obtained concentration and path length according to Equation (1). In this retrieval process, gas concentration in all segments can be obtained to achieve the measurement of gas distribution along the whole target path. The results of the fitting for the different absorption lines which were derived from the IGM that returned from Seg.2 are shown in Figure 6a–c. The measured mean equivalent concentration *α*_ref_ and *α*_seg1_ is zero, and *α*_seg2_ is 1.71 mol∙L^−1^. According to the equivalent relationship *α*_cell_ × *L*_cell_ = *α*_seg2_ × *L*_seg2_ (nominal length *L*_cell_, 7.5 cm), the gas concentration *α*_cell_ in the cell is calculated as 5.32 mol∙L^-1^. In this benchtop experiment, *L*_cell_ can be directly used for fitting to retrieve the *α*_cell_. The retrieval of equivalent concentration used in the above conversion is aim to demonstrate the calculation process of the proposed method in practical sensing applications. The pressure in the cell converted from *α*_cell_ is 98.92 torr, and the relative deviation from the nominal pressure (100 ± 0.2 torr) is 1.08%. It can be seen that segment-resolved gas concentration measurement can effectively help to determine the gas distribution and then locate the concentration change. Unfortunately, due to the lack of line parameters for H^13^CN and ^13^C_2_H_2_ in the measurement range, we failed to obtain the equivalent gas concentrations of these two gas types. Here, we only show the absorption lines of H^13^CN and ^13^C_2_H_2_ from the three different spectra in Figure 6d,e to demonstrate the possibility of concentration retrieval and the good consistency of spectral measurement for the same gas.

## 5. Discussion

The measurement uncertainty of gas concentration is a general concern for various applications and can be mainly attributed to two sources: the measurement uncertainties of the absorption spectrum and segmented path length. On the absorption spectrum side, the SNR, multiplexing crosstalk and fitting residual are the main sources of uncertainty. Among them, the multiplexing crosstalk is a special term in the proposed method. To evaluate their contributions, the Monte-Carlo method was used [33] and the values of 400:1 (in 1 s) for SNR, 0.02% (ZPD interval is about 40 cm) for the multiplexing crosstalk and 0.02% for the spectral fitting residual were adopted in the evaluation. The calculated relative uncertainty of gas concentration retrieval introduced by them is 0.7% and the contribution from the multiplexing crosstalk is very small. On the path length side, envelope peak positioning, gas refractive index and optical components in the path are the main sources of uncertainty. Here, we first evaluated their values for the uncertainty of path length. The uncertainty induced by the peak positioning method is evaluated as 7.2 × 10^−7^ m based on the Monte-Carlo method [34] considering sampling fluctuation and noise. The uncertainty induced by the gas refractive index for standard atmosphere is evaluated as 2.2 × 10^−7^ m, considering the fluctuations of the environmental parameters, such as 0.5 K for temperature, 26.6 Pa for pressure, and 1.2% for humidity. Because it is a paradox to accurately estimate the refractive index of sample gas before knowing its concentration, we enlarged the uncertainty component value by three times (6.6 × 10^−7^ m) for practice. The optical components in the path induced an uncertainty of 1.3 × 10^−4^ m in such a condition that the nominal error limit of the component length is 0.05 mm and that of the glass refractive index is 5 × 10^−5^. The relative uncertainty of path length introduced by all these sources is 2.8 × 10^−4^, and the corresponding relative uncertainty of concentration retrieval is less than 3 × 10^−4^, which is one order smaller than that from the spectrum. So in such spectroscopy application, the measurement uncertainty of path length is not as critical as in other ranging applications.

The dual-comb method has attracted much attention in gas remote sensing research, in which the monitoring of gas concentration throughout the selected region was enabled in the simulation of potential leakage sources. However, it is essentially based on the concentration measurement of the multiple independent paths to infer whether the source was leaking. It is believed that gridded gas concentration changes can provide more accurate and efficient information. Since the gas concentration in each path-segment along a single target path can be obtained by this proposed method, it can be inferred that the gas distribution in the whole region can be further gridded with the combination of multiple paths. The potential capability of dual-comb method is well suited to meet the further needs for gas sensing. Our experiment is just the pre-validation of such a distributed remote sensing (DRS) method for gridded measurement of the regional gas concentration. If this method can really be performed in the open-path measurement, the large aperture of the beam can be used for splitting to improve the utilization efficiency of light and dual-comb method can more fully exert its excellent performance, such as rapid data update speed and direct turbulence immunity, to meet the challenges of the rapid environmental changes.

## 6. Conclusions

We propose a gas distribution measurement method to locate the gas concentration changes on each segment along the target path, which simultaneously combines the measurement capabilities of dual-comb method in the field of spectroscopy and ranging. The feasibility of this time domain multiplexed dual-comb gas detection method has been verified in benchtop experiments with the results of absorption spectrum measurement, path length measurement, and further concentration retrieval. The equivalent gas concentration in each path-segment can be retrieved separately with a relative deviation of 1.08%. Gas distribution measurement for the target path can be achieved with the segment-resolved gas concentration. It is also proved in theory that the crosstalk between the interference signals has only an effect of no more than 0.1% in the 1000 m open-path atmospheric absorption measurement. The gas monitoring process will be pushed from line to surface if this method can be used to realize the gridding of regional gas concentration measurement, which is expected to meet further actual application requirements.
